# Supplementary material for: The association between subfoveal choroidal thickness and refractive error in Taiwanese children: A cross-sectional study
Source: PLoS One. 2025 May 19;20(5):e0316306. doi: 10.1371/journal.pone.0316306 (PMC12088011; doi:10.1371/journal.pone.0316306)
Supplement: Attachment — Submitted filename: Response to Reviewers.docx [file pone.0316306.s002.docx]

**Response to Reviewers**

Dear Editor, we appreciate you and the reviewers for your precious time in reviewing our paper and providing valuable comments. It was your valuable and insightful comments that led to possible improvements in the current version. We have carefully considered the comments and tried our best to address every one of them. We hope the manuscript after careful revisions meet your high standards. The authors welcome further constructive comments if any. Below we provide the point-by-point responses. All page numbers refer to the revised manuscript file with tracked changes.

Reviewer #1

The manuscript explores the association of subfoveal choroidal thickness (SFCT) with various ocular and demographic factors in Taiwanese children, an important area considering the high prevalence of myopia in Asia. While the study adds value, particularly in a pediatric Taiwanese cohort, several areas require clarification or improvement.
**Author response:** Thank you very much for your comments that helped us improve this manuscript.
Title
1. Title Clarity: Consider revising the title to "The Association Between Subfoveal Choroidal Thickness and Refractive Error in Taiwanese Children: A Cross-sectional Study and Systematic Review." This emphasizes the study's primary focus and methods.
**Author response:** As suggested by the reviewer, we have revised the title to “The Association Between Subfoveal Choroidal Thickness and Refractive Error in Taiwanese Children: A Cross-sectional Study."
Abstract
1. Methods: The abstract should explicitly include the study's design (e.g., "retrospective cross-sectional study").

**Author response:** Thanks for your kind reminders. We have added “retrospective” into the sentence as follos:
“A total of 374 eyes in 187 children were enrolled in this retrospective cross-sectional comparative study” (Pg 1, Ln 28-29)
2. Results: The key findings are clear but could emphasize multivariate analysis outcomes, as they are more robust.

**Author response:** Thanks for your kind reminders. We revised the sentence as follows:

“However, after adjusting in the multivariate regression analysis, spherical equivalent, sphere power and age were not independently associated with SFCT. In multivariate analysis, lower cylinder power and longer axial length have significant correlations with thinner SFCT.” (Pg 1, Ln 39-42)
3. Conclusion: Avoid speculative phrases like "might be a good predictor." Instead, state the significance of findings confidently while reserving speculation for the discussion.
**Author response:** Thanks for your kind reminders. We revised the sentence as follows:

“This study showed that mean subfoveal choroidal thickness was 299.0 ± 69.80 μm among Taiwanese children., The SFCT was thinner in myopic, longer axial length, and lower cylinder power eyes.” (Pg 1, Ln 42-44)
Introduction
1. Literature Context: Expand on how the study differs from previous works in Chinese, Korean, or Australian children to establish the novelty better.

**Author response:** Thanks for your kind reminders. We revised the sentence in this part as follows:
“Due to high prevalence of myopia in Asia area, there had been many researches about it in Singapore, China, and South Korea. A study from Singapore, Gupta et al. reported extremely high myopic eyes had thinner choroid [13]. The mean AL was 23.65±0.89 mm and the mean age of their study was 21.96±0.89 years. A study from Japan that was conducted by Fujiwara et al. reported SFCT depends on age and refractive error [5]. A study from Korea, Lee et al. found choroidal thickness were greater in the hyperopic populations than in the emmetropic and myopic populations [4]. A study from China, Zhu et al. investigated the 52 Chinese children, they reported the thicker SFCT in hyperopia children, and correlated with shorter axial and higher spherical equivalent [9]. Most studies consistently showed that higher myopia is associated with thinner SFCT. However, these studies covered different age groups and had inconsistent results about choroidal thickness and other ocular characteristics. Studies on children are also rare, with most studies focusing on young adults. Furthermore, there have been limited data of the choroidal thickness in Taiwanese children. Due to above, we aimed to analyze the association of SFCT with factors including age, best-corrected visual acuity, refractive error, and axial length in Taiwan pediatric population; also compared with different countries.” (Pg 2-3, Ln 69-83)
We also had more detailed discussion at Pg 10-11, Ln 182-204.
2. Aim Clarity: The aim is well stated, but the introduction would benefit from explicitly linking SFCT to its clinical implications in predicting myopia progression.
**Author response:** Thanks for your kind reminders. Our study is retrospective designed; therefore, we cannot explicitly link SFCT to predict myopia progression.
Methods
1. Study Design: Clarify why a retrospective design was chosen and discuss the potential limitations of retrospective data.

**Author response:** We choose retrospective designed study because of we can get most patient data in a short period. The limitations of retrospective designed including incomplete data and we cannot approve the predictivity of subfoveal choroidal thickness and myopia progression. (Pg 14, Ln 282-284)
2. Definitions: Provide more details about cycloplegic refraction procedures (e.g., drugs used, cycloplegic protocol).

**Author response:** Thanks for your kind reminders. We have revised accordingly. (Pg 4, Ln 97-99)
3. Exclusions: Add rationale for excluding patients with incomplete exams or prior myopia control treatments.

**Author response:** Thanks for your kind reminders. We have revised the sentence as follows:

“ Patient who had previously diagnosed retinal pathology, received myopia control treatment or incomplete examination were excluded to prevent interference results.” (Pg 4, Ln 93-95)
4. Measurement: Mention the interobserver variability or agreement for manual SFCT measurements.
**Author response:** Thanks for your kind reminders. We have revised the sentence as follows:

“All measurements were taken by two experienced ophthalmologists simultaneously, who were masked to the other parameters of the patient. Only the measurements results agreed by both ophthalmologists will be recorded.” (Pg 4, Ln 104-107)
Results
1. Demographics: The mean age and axial length provide useful context, but gender-based differences in axial length (significant) and SFCT (non-significant) should be discussed more comprehensively.

**Author response:** Thanks for your kind reminders. We had discussed more comprehensively as follows:
“Our study demonstrated that male had more myopia and longer axial length. Although, there was no significant difference of SFCT between genders, male had thinner mean SFCT, which is corresponded to higher myopia and longer axial length in males. Some studies reported that female had thinner SFCT, but others reported no significant differences between boys and girls for SFCT and SER [3,6,10]. Cevher et al. reported females have higher basal sympathetic tone than males which can cause vasoconstriction and induced thinner choroidal thickness [10]. In our studies, mean age is 5-years-old, which is too young to have significant higher sympathetic tone. This may explain the difference between our study and other studies.” (Pg 11, Ln 214-221)
2. Regression Analysis: The multivariate analysis results should be emphasized more, as they account for confounders. Consider rephrasing for clarity.
**Author response:** Thanks for your kind reminders. We had rephrasing and emphasized more clarity in result and discussion. (Pg 8, Ln 158-162; Pg 13, Ln 254-259)
Discussion
1. Comparison with Literature: The study compares findings with data from other regions but needs to delve deeper into why SFCT in Taiwanese children aligns more closely with other Asian cohorts than non-Asian ones.

**Author response:** Thanks for your kind reminders. The choroidal thickness in different ethnicities had been found significant difference. Youngseok Song et al. noted Malays exhibited thinner SFCT compared with Indians and Chinese. Rhodes et al also reported choroidal thickness varies with race, that African descent have a thicker peripapillary choroid than European descent. Although there is no date compare the choroidal thickness between Asian and Europeans, the SFCT between Asians will be more close than non-Asian ones.

We added these contents into Discussion. (Pg 12-13, Ln 234-239)
2. Clinical Implications: Discuss how these findings could influence myopia management in children (e.g., early interventions for thinner SFCT).

**Author response:** Thanks for your kind advise. We had revised the sentence as follows:

“ Our findings provide the profiles of SFCT in the eyes of Taiwanese children populations and to offer insights into the early interventions and monitors for thinner SFCT and lower astigmatism.” (Pg 15, Ln 348-349)
3. Cylindrical Power: The association of SFCT with cylindrical power is novel but requires more discussion. Could this be a surrogate for corneal or lenticular changes in myopic eyes?

**Author response:** It is a good question. As we mentioned in our prescription, the association of cylinder and SFCT had not been mentioned in previous reports. Children with higher astigmatism had been reported to have more HOAs, which exhibited significant correlations with reduced myopia progression and axial elongation. Based on above, higher cylinder power is associated with higher HOA, which reduced axial elongation and myopia progression, might have thicker SFCT. The result still needs to be confirmed by more longitudinal studies. (Pg 13-14, Ln 260-268)
4. Limitations: The discussion acknowledges the lack of longitudinal data and manual SFCT measurements but should also address potential selection bias in a hospital-based cohort.

**Author response:** Thanks for your kind advise. This hospital-based cohort might have selection bias due to patient might have some ocular disease or use medication treatments. Therefore, we excluded patient who had previously diagnosed retinal pathology, received myopia control treatment. However, the hospitals in Taiwan are highly accessible and most Taiwanese children are likely to receive ocular examination and visual screening since preschool age due to the national policy. (Pg 14-15, Ln 342-346)

Reviewer #2: Comments to the Editor of PLOS ONE
Dear Editor,
Thank you for the opportunity to review this manuscript. Please find my comments below.
**Introduction**
The manuscript does not provide a compelling justification for the need of the study. It would benefit from a more robust rationale for why the research is important. Additionally, the background section should better explain the potential relationship between choroidal thickness, myopia, and other relevant parameters measured in the study.

**Author response:** Thanks for your comment. This is the first Taiwanese children population study for SFCT and associated characteristics and ocular factors. Furthermore, we found the thinner SFCT was correlated with older age, myopia, longer axial length, and lower cylinder power. This is also the first study reported the association between cylinder power and SFCT. We had revised as accordingly as follows:
“Due to high prevalence of myopia in Asia area, there had been many researches about it in Singapore, China, and South Korea. A study from Singapore, Gupta et al. reported extremely high myopic eyes had thinner choroid [10]. The mean AL was 23.65±0.89 mm and the mean age of their study was 21.96±0.89 years. A study conducted in Australia investigated the 104 children with mean age 13.1± 1.4 years [15]. This study reported myopic children have thinner choroids thickness, particularly in central foveal regions. A study from Japan that was conducted by Fujiwara et al. reported subfoveal choroidal thickness depends on age and refractive error [5]. A study from Korea, Lee et al. found choroidal thickness were greater in the hyperopic populations than in the emmetropic and myopic populations [4]. A study from China, Zhu et al. investigated the 52 Chinese children, they reported the thicker subfoveal CT in hyperopia children, and correlated with shorter axial and higher spherical equivalent [9]. Most studies consistently showed that axial length is associated with SFCT. However, these studies covered different age groups and had inconsistent results about choroidal thickness and ocular characteristics. Studies on children are also rare, with most studies focusing on young adults. Furthermore, there have been limited data of the choroidal thickness in Taiwanese children. Due to above, we aimed to analyze the association of subfoveal choroidal thickness (SFCT) with factors including age, best-corrected visual acuity, refractive error, and axial length in Taiwan pediatric population; also compared with different countries.” (Pg 2-3, Ln 69-83)
**Methods**
The criteria for including and excluding participants in the study were not sufficiently specified. It is important to provide clearer and more detailed information on the selection process. Furthermore, a more thorough description of how the clinical assessments were conducted is necessary to ensure transparency and reproducibility.
**Author response:** Thanks for your kind advise. We had revised as follows:
“Medical records of Children under 16-years-old visited Ophthalmic center in China medical university hospital from 01/08/2019 to 08/31/2022 were included. The data used in this study were accessed between 03/20/2023 and 03/19/2024. Patient who had previously diagnosed retinal pathology, received myopia control treatment or incomplete examination were excluded to prevent interference results and selection bias.” (Pg 4, Ln 90-95)
**Discussion**
The discussion section is lacking a comprehensive analysis of the study's findings in comparison with previous research. It would be valuable to include a comparison with existing literature, noting any similarities or discrepancies, and providing potential explanations for these observations. Furthermore, the discussion should be revised to avoid redundancy and repetition of the results section.

**Author response:** Thanks for your kind advise. We had revised accordingly. (Pg 10-11, Ln 182-204)
Strengths and Limitations
While the manuscript addresses some limitations of the study, it does not mention its strengths. It would be helpful to provide a more balanced view by discussing the study’s strengths in addition to its limitations.

**Author response:** Thanks for your kind advise. We had revised as follows:
“ Our study had several strengths. First, this is the first Taiwanese children population study exploring the association of choroidal thickness. Second, the results of the association of cylindrical power and SFCT was first reported. Third, we had excluded children with ocular disease or received atropine treatment to less selection bias.” (Pg 14, Ln 275-278)
Recommendation
Overall, the study presents some merits. However, I recommend a major revision to address the points outlined above

**Author response:** Thank you for your comments. We have gone through your comments carefully and tried our best to address them one by one. We hope the manuscript has been improved accordingly.
